# Rapid mitochondrial repolarization upon reperfusion after cardiac ischemia

**DOI:** 10.1038/s44161-025-00752-9

**Published:** 2025-12-11

**Authors:** Abigail V. Giles, Raul Covian, Hiran A. Prag, Nils Burger, Bertrand Lucotte, Chak Shun Yu, Junhui Sun, Elizabeth Murphy, Thomas Krieg, Michael P. Murphy, Robert S. Balaban

**Affiliations:** 1 NHLBI, National Institutes of Health, Bethesda, MD USA; 2 MRC Mitochondrial Biology Unit, University of Cambridge, Cambridge, UK; 3 Department of Medicine, University of Cambridge, Cambridge, UK

**Keywords:** Biophysics, Cardiovascular biology

## Abstract

The mitochondrial membrane potential (ΔΨ_m_) drives oxidative phosphorylation and alterations contribute to cardiac pathologies, but real-time assessment of ΔΨ_m_ has not been possible. Here we describe noninvasive measurements using mitochondrial heme *b*_L_ and *b*_H_ absorbances, which rapidly respond to ΔΨ_m_. Multi-wavelength absorbance spectroscopy enabled their continuous monitoring in isolated mitochondria and the perfused heart. Calibration of heme *b* absorbance in isolated mitochondria revealed that reduced heme *b*_L_ relative to total reduced heme *b* (f*b*_L_ = *b*_L_/(*b*_L_ + *b*_H_)) exhibits a sigmoidal relationship with ΔΨ_m_. Extrapolating this relationship to the heart enabled estimation of ΔΨ_m_ as 166 ± 18 mV (*n* = 25, mean ± s.d.). We used this approach to assess how ΔΨ_m_ changes during ischemia–reperfusion injury, an unknown limiting the understanding of ischemia–reperfusion injury. In perfused hearts, ΔΨ_m_ declined during ischemia and rapidly reestablished upon reperfusion, supported by oxidation of the succinate accumulated during ischemia. These findings expand our understanding of ischemia–reperfusion injury.

## Abstract (web summary)

Giles et al. developed a method for noninvasive absorbance measurement of mitochondrial hemes to monitor the mitochondrial membrane potential in the perfused heart. They then applied this approach to show how the mitochondrial membrane potential changed during cardiac ischemia.

## Main

**Fig. 1:**
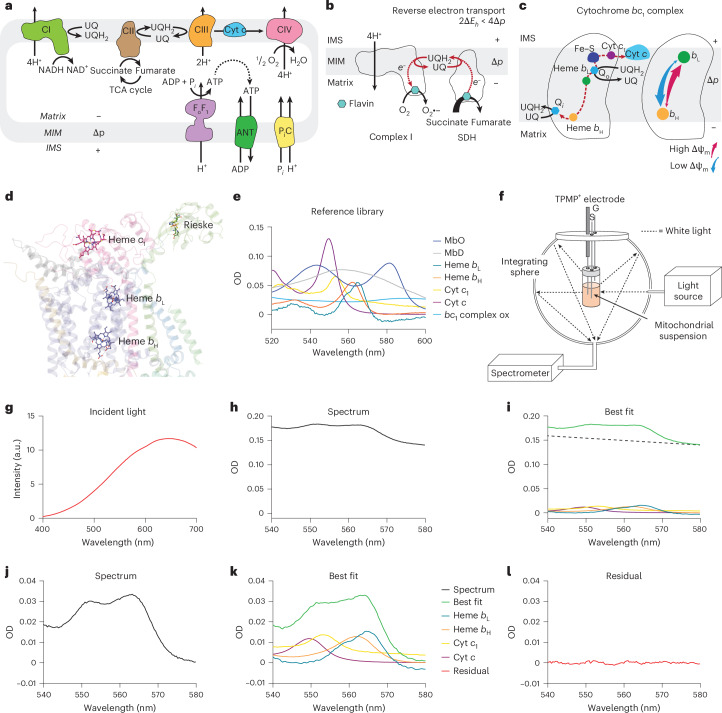
Multi-wavelength absorbance spectroscopy in isolated mitochondria. **a**, Mitochondrial oxidative phosphorylation. **b**, Upon reperfusion, succinate accumulated during ischemia is proposed to be oxidized by SDH, driving RET and superoxide production initiating ischemia–reperfusion injury. IMS, intermembrane mitochondrial space; MIM, mitochondrial inner membrane. **c**, Electron transfer within the cytochrome *bc*_1_ complex and electron distribution between hemes *b*_L_ and *b*_H_ in response to ΔΨ_m_. **d**, Structure of the bovine cytochrome *bc*_1_ complex from PDB 1BE3. The distinct coordination environments of the heme groups in the *bc*_1_ complex give rise to unique absorption spectra. **e**, Reference spectra including for reduced hemes *b*_L_ and *b*_H_ and cytochromes *c*_1_ and *c* and for oxygenated (MbO) and deoxygenated (MbD) purified mouse myoglobin, as well as oxidized cytochrome *bc*_1_ complex, were collected using a combination of biochemical techniques. Thus, relative optical density (OD) is arbitrary. **f**, Schematic of the integrating sphere system used to measure the optical absorbance of mitochondrial suspensions. TPMP^+^ distribution was measured simultaneously by a TPMP^+^-selective electrode. Substrates, inhibitors and uncouplers (S) and gases (G) were introduced via separate ports. **g**, Spectrum of incident light used to analyze mitochondrial suspensions. a.u., arbitrary units. **h**, Isolated mitochondria were incubated with succinate/rotenone, and a multi-wavelength nonlinear least squares regression was performed on the experimental absorbance spectrum using reference spectra for mitochondrial hemes and cytochromes and a line. **i**, The best fit and individual chromophore contributions. **j**,**k**, The linear component, incorporated into the fit to account for absorption by the integrating sphere and oxidized chromophores, was subtracted from the experimental spectrum (**j**) and best fit (**k**). **l**, The residuals of the best fit. Source data

During mitochondrial oxidative phosphorylation (Fig. 1a), the respiratory chain pumps protons across the mitochondrial inner membrane to establish a protonmotive force (Δ*p*), composed of a large electrical potential gradient (ΔΨ, negative inside) and a smaller pH gradient (ΔpH, basic inside)^1,2^. The Δ*p* drives adenosine triphosphate (ATP) synthesis and export to the cytosol, ion transport, protein import and thermogenesis, as well as the production of superoxide (O_2_^•−^), which is the proximal reactive oxygen species (ROS) in many pathologies (Fig. 1b)^2,3^. In the heart, ATP production is largely driven by oxidative phosphorylation^4^, but the magnitude of the mitochondrial membrane potential (ΔΨ_m_) (the dominant component of Δ*p*^2^) remains unclear. Furthermore, ΔΨ_m_ is thought to have a key role in several aspects of cardiac ischemia–reperfusion injury including the production of ROS (Fig. 1b)^1,5^, the capacity to produce ATP and mitochondrial calcium handling. However, it has not been possible to noninvasively measure ΔΨ_m_ in the intact heart during ischemia–reperfusion injury. To address these critical, unresolved issues, here we quantified rapid changes in ΔΨ_m_ in real time within the isolated perfused mouse heart subjected to global ischemia–reperfusion using endogenous chromophores.

## Results

### Quantification of ΔΨ_m_ using *b* hemes

Lipophilic cations are accumulated within the mitochondria in response to ΔΨ_m_ and the plasma membrane potential (ΔΨ_p_) in accordance with the Nernst equation and, thus, are commonly used for determining ΔΨ_m_ in vitro^6,7^. Changes in ΔΨ_m_ within cells can be inferred from the distribution of fluorescent lipophilic cations including tetramethylrhodamine methyl ester (TMRM). Ion-selective electrodes can be used to determine ΔΨ_m_ in suspensions of isolated mitochondria incubated with lipophilic cations such as triphenylmethylphosphonium (TPMP^+^)^8^. Non-optical approaches with potential application in vivo include assessing the distribution of radiolabeled lipophilic cations^9–11^ or direct visualization of positron emission tomography tracers incorporated into lipophilic cations^12,13^. Optical approaches for assessing mitochondrial activity that have been applied to the isolated perfused heart include assessing the redox state of NAD(P)H and flavoprotein pools by autofluorescence^14,15^ and monitoring the fluorescence of lipophilic cationic dyes such as safranine^16^ or TMRM^17^. However, the application of lipophilic probes and dyes is challenging in the intact, perfused heart due to uncertainty about the uptake, binding and distribution of these probes^18^. Furthermore, using these approaches to quantify ΔΨ_m_ relies on multiple assumptions^8^, while the slow equilibration of lipophilic cations within tissues^19^ precludes the rapid assessment of ΔΨ_m_ changes that is required to understand how ΔΨ_m_ responds to ischemia–reperfusion injury.

To overcome these limitations, here we quantified ΔΨ_m_ in real time using endogenous chromophores. The mitochondrial cytochrome *bc*_1_ complex contains *b*_L_ (lower reduction potential, *E*_m_ = –90 mV (ref. ^20^)) and *b*_H_ (higher reduction potential, *E*_m_ = –30 mV (ref. ^20^)) hemes positioned close to the positive and negative sides of the inner membrane, respectively (Fig. 1c)^21^. During respiration, electrons are reversibly transferred from *b*_L_ to *b*_H_, which also exchanges electrons with the coenzyme Q (CoQ) pool. The exchange of electrons between *b*_L_ and *b*_H_ depends on the relative reduction potential of these hemes and on ΔΨ_m_, with a decrease in ΔΨ_m_ favoring electron migration from *b*_L_ to *b*_H_ (Fig. 1c). Thus, the amount of reduced *b*_L_ relative to reduced *b*_H_ provides insight into ΔΨ_m_. The optically distinct absorbance of each *b* heme increases in the reduced state, so as ΔΨ_m_ rises, the absorbance of *b*_L_ is expected to increase relative to that of *b*_H_. To compensate for the changes in the redox state of the cytochrome *bc*_1_ complex due solely to differences in respiration rate or substrate availability, the fraction of reduced heme *b*_L_ should be normalized to the amount of total reduced *b* heme (*b*_L_ + *b*_H_). Following this approach, we generated a parameter f*b*_L_ = *b*_L_/(*b*_L_ + *b*_H_) that is highly sensitive to ΔΨ_m_ and can be calculated from the absorbance of the *b*_L_ and *b*_H_ hemes. This approach is possible because the distinct coordination environments of the heme groups in the *bc*_1_ complex (Fig. 1d) give rise to unique absorbance spectra, enabling multi-wavelength absorbance spectroscopy. Hemes *b*_H_ and *b*_L_ are spectrally distinct with absorption peaks at 562 nm and 564 nm, respectively (Fig. 1e). Over the bandwidth of interest where the reduced hemes of the cytochrome *bc*_1_ complex absorb (540–580 nm), there is also absorption by reduced cytochrome *c* and cytochrome *c*_1_ (Fig. 1e). Reduced cytochrome *c* has peak absorption at 550 nm, whereas reduced cytochrome *c*_1_ (*E*_m_ = 230 mV^22^) is slightly red shifted with peak absorption at 552 nm.

The relationship between *b*_L_/*b*_H_ reduction and ΔΨ_m_ was established in submitochondrial particles^23^ and cytochrome *bc*_1_ reconstituted into liposomes^24^, with this relationship later extrapolated to cultured cells to estimate ΔΨ_m_ (ref. ^25^). While promising in principle, optical analysis in cells was limited because the calculation of ΔΨ_m_ was inferred from midpoint reduction potential (*E*°) values for the *b* hemes obtained in vitro^26^. Therefore, to use this method to quantify ΔΨ_m_ in the intact, perfused heart from the reduction states of *b*_L_ and *b*_H_, f*b*_L_ has to be calibrated against an orthogonal measure of ΔΨ_m_, to avoid using *E*° values generated under nonphysiological conditions.

### Calibrating *b* heme absorbance against ΔΨ_m_

While optical methods have been used to study mitochondrial cytochromes for many decades^27,28^, quantification of cytochrome reduction status has usually relied on dual-wavelength analysis in which absorbance peaks are measured relative to absorbance minima or putative isosbestic points^29^. However, this approach is unreliable, particularly in complex systems such as the intact heart, due to the overlapping spectral features of biochemically independent chromophores^30–32^. Consequently, dual-wavelength analysis has been superseded by multi-wavelength absorbance spectroscopy and spectral analysis, which has been applied to isolated mitochondria and the perfused heart^30–32^. The advantage of using multi-wavelength spectral fitting is that the spectral density at all wavelengths is used to determine the absorption of individual cytochromes, rather than relying on single peak absorption wavelengths, which greatly facilitates the deconvolution of spectrally similar cytochromes, enabling robust determination of *b*_L_ and *b*_H_ absorbance. Here, we used multi-wavelength absorbance spectroscopy to determine the reduction state of mitochondrial *b* hemes within both isolated mitochondria and the perfused mouse heart.

To determine the absorbance of *b*_L_ and *b*_H_, and thus f*b*_L_, in isolated mitochondria, the optical absorbance of heart mitochondria was determined using an integrating sphere system optimized to capture light scattered by the turbid suspension (Fig. 1f) and then fit with reference spectra for reduced (Fe^2+^) cytochromes (Fig. 1e). This reference library was generated in the integrating sphere system using a combination of biochemical techniques, which are described in detail in the Methods section. For this reason, the optical density of the reference spectra does not reflect absolute absorption. A white light source (Fig. 1g) was passed through the sample. Experimental absorbance spectra (Fig. 1h) from 540 to 580 nm were fit with reference spectra representing reduced heme *b*_H_, heme *b*_L_, cytochrome *c*_1_ and cytochrome *c* using a nonlinear least squares regression (Fig. 1i), enabling accurate determination of the absorbance of individual mitochondrial chromophores^30–33^. Our analysis includes a linear component with a variable slope (Fig. 1i)^34,35^ to account for residual scattering and the weak absorbance by oxidized (Fe^3+^) cytochromes, which lack spectral definition and have a lower optical density relative to the reduced hemes over this spectral range (Fig. 1e)^35^. To facilitate visualization of mitochondrial cytochromes, the linear component was subsequently subtracted from the experimental spectrum and best fit (Fig. 1j,k). Residuals, calculated as the difference between the experimental spectrum and the spectral fit, were minimal, demonstrating a good fit (Fig. 1l).

**Fig. 2:**
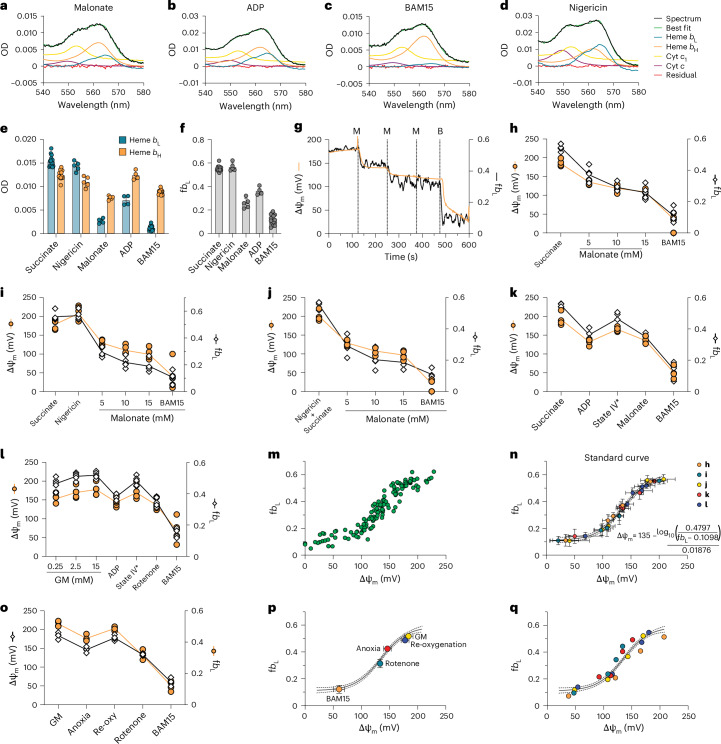
In vitro calibration of *b* heme absorbance against ΔΨ_m_. **a**–**d**, Experimental absorbance spectra and best fits of mitochondria incubated with succinate/rotenone and 30 mM malonate (**a**), ADP (**b**), BAM15 (**c**) or nigericin (**d**). After spectral fitting, the linear component was subtracted from all spectra and best fits. **e**, Mitochondria were incubated with succinate/rotenone (*n* = 14) and nigericin (*n* = 5), 30 mM malonate (*n* = 5) or ADP (*n* = 4). BAM15 was added at the conclusion of all experiments (*n* = 14). Heme *b*_L_ and *b*_H_ absorbance was quantified from corresponding spectral fits. **f**, f*b*_L_ was calculated from *b* heme absorbance. **g**, Representative time course of mitochondria incubated with succinate/rotenone and titrated with malonate (M) and BAM15 (B). A rolling average of five consecutive spectra was taken before multi-wavelength nonlinear least squares regression analysis and quantification of heme *b*_L_ and *b*_H_. ΔΨ_m_, calculated from the Nernstian distribution of TPMP^+^, and f*b*_L_ are plotted over time. **h**, f*b*_L_ and ΔΨ_m_ under each condition (*n* = 5 biological replicates). **i**–**l**, Mitochondria were incubated sequentially with succinate, nigericin, malonate and BAM15 (**i**); succinate and nigericin, malonate and BAM15 (**j**); succinate, ADP, malonate and BAM15 (**k**); and GM ADP, rotenone and BAM15 (**l**). f*b*_*L*_ and ΔΨ_m_ were determined under each condition shown. A line connects mean values. **m**, f*b*_L_ plotted as a function of ΔΨ_m_ for all data points derived from **h**–**l**. **n**, Average f*b*_L_ plotted as a function of average ΔΨ_m_ for all conditions shown in **h**–**l** (*n* = 23). Data were fit using nonlinear regression with a four-parameter sigmoidal model with variable slope (solid line), and 95% confidence intervals were calculated (dotted lines). **o**, Mitochondria were incubated with GM and deoxygenated by blowing N_2_ over the suspension before reoxygenating 5 min later with O_2_. A line connects mean values. **p**, f*b*_L_ and ΔΨ_m_ were determined at each steady state shown (*n* = 5). f*b*_L_ is plotted as a function of ΔΨ_m_ and superimposed onto the sigmoidal curve generated in **n**. **q**, Mouse heart mitochondria were incubated with GM followed by ADP, rotenone and BAM15, and f*b*_L_ and ΔΨ_m_ were determined under each condition (*n* = 5). These data are superimposed onto the sigmoidal curve generated in **n**. Unique colors are used to represent biological replicates. All data are shown as the mean ± s.d. Source data

The absorbance amplitudes of hemes *b*_H_ and *b*_L_ were similar in energized mitochondria, where ΔΨ_m_ is close to maximum (Fig. 1k). When ΔΨ_m_ was decreased by addition of the succinate dehydrogenase (SDH) inhibitor malonate, ADP or the uncoupler BAM15, *b*_L_ became more oxidized relative to *b*_H_, consistent with a decrease in ΔΨ_m_, whereas the absolute absorbance by the *b* hemes was changed little by nigericin, which has a minor effect on ΔΨ_m_ (Fig. 2a–e). Thus *b*_L_ and *b*_H_ change as anticipated in response to alterations in ΔΨ_m_ in isolated mitochondria, and the derived parameter f*b*_L_accurately reflects anticipated changes in ΔΨ_m_ (Fig. 2f).

To use f*b*_L_ to quantify ΔΨ_m_ in biological systems, f*b*_L_ must be calibrated against an orthogonal determination of ΔΨ_m_. We measured the distribution of the lipophilic TPMP^+^ cation across the mitochondrial inner membrane using an ion-selective electrode to precisely quantify ΔΨ_m_, a long-established approach for determining ΔΨ_m_ in vitro^8^. Simultaneously, we measured *b*_L_ and *b*_H_ absorbance. Mitochondria inhibited with rotenone were initially fully energized with succinate and then titrated with malonate to sequentially lower ΔΨ_m_, followed by addition of the uncoupler BAM15, while f*b*_L_ and ΔΨ_m_ were measured concurrently (Fig. 2g,h). To determine whether matrix pH affected the relationship between f*b*_L_ and ΔΨ_m_, mitochondria respiring on succinate were treated with the ionophore nigericin, which abolishes the pH gradient, and titrated as described above (Fig. 2d,i,j). To further interrogate the relationship between f*b*_L_ and ΔΨ_m_, mitochondria energized with succinate received a bolus of ADP to decrease ΔΨ_m_ to a lower steady-state value, before uncoupling with BAM15 (Fig. 2k). When glutamate-malate (GM) was used as an NADH-linked electron donor, as opposed to FADH-linked succinate, ΔΨ_m_ was similarly decreased with ADP (Fig. 2l). These disparate experiments enabled a calibration curve of ΔΨ_m_ against f*b*_L_ to be constructed (Fig. 2m,n), showing a monotonic relationship between f*b*_L_ and ΔΨ_m_ over a wide range of conditions. The sigmoidal shape of this calibration curve will make the estimation of ΔΨ_m_ less certain at high and low levels where the change in f*b*_L_ is small relative to the change in ΔΨ_m_. A similar relationship was observed when tetraphenylphosphonium (TPP^+^) was used instead of TPMP^+^, suggesting that either cation can be used to monitor ΔΨ_m_. To determine whether the relationship between f*b*_L_ and ΔΨ_m_ is valid when the mitochondrial respiratory chain is reduced by anoxia, mitochondria incubated with GM were subjected to anoxia–reoxygenation (Fig. 2o). The unique relationship between f*b*_L_ and ΔΨ_m_ was retained during anoxia (Fig. 2p) and in the presence of nigericin, which lowers the pH of the mitochondrial matrix, similar to what occurs during ischemia (Fig. 2j). Although we elected to use rabbit mitochondria, due to the amount of material required to execute these studies, we also investigated the relationship between f*b*_L_ and ΔΨ_m_ in mouse heart mitochondria. We found that the calibration of f*b*_L_ to ΔΨ_m_ in the mitochondria from rabbit and mouse was indistinguishable (Fig. 2q), enabling the translation of this approach to the isolated mouse heart model.

In summary, f*b*_L_ determined by optical spectroscopy is a normalized measure that responds solely to changes in ΔΨ_m_ under a range of conditions, including anoxia. This development enables assessment of ΔΨ_m_ in models where heme *b*_L_and *b*_H_ absorbance can be determined, including the isolated perfused heart.

### Measurement of ΔΨ_m_ in the isolated heart

**Fig. 3:**
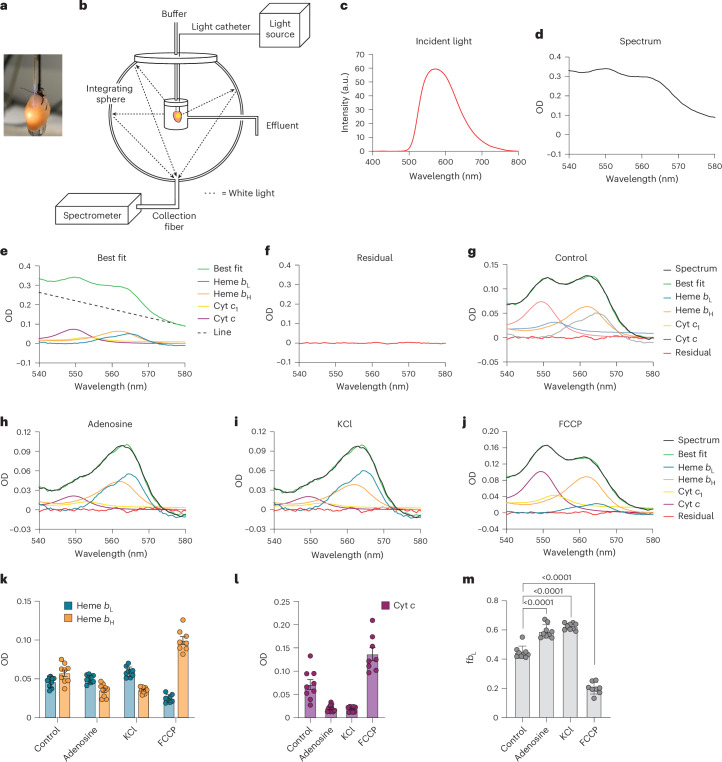
Measurement of ΔΨ_m_ in the heart. **a**, Photograph of an isolated, perfused mouse heart with an optical light catheter (200 µm) inserted through the mitral valve and positioned in the left ventricle. **b**, Schematic of the integrating sphere system used to measure light transmittance across the left ventricular wall in real time. **c**, Incident light spectrum. **d**, The isolated perfused *Mb*^−/−^ mouse heart was perfused for 20 min, and a multi-wavelength nonlinear least squares regression was performed on the experimental spectrum using reference spectra for mitochondrial hemes and cytochromes and a line. **e**, The best fit and individual chromophore contributions. **f**, The residuals of the best fit. **g**, Data in **d**–**f** with the linear component subtracted from the experimental spectrum and best fit. **h**–**j**, Isolated perfused hearts from *Mb*^−/−^ mice were treated in sequence with adenosine (**h**), KCl (**i**) and FCCP (**j**) to alter ΔΨ_m_. Absorbance spectra were fit by multi-wavelength nonlinear least squares regression as described above. After spectral fitting, the linear component was subtracted from all experimental spectra and best fits. **k**,**l**, Heme *b*_L_ and *b*_H_ (**k**) and cytochrome *c* (**l**) absorbances were quantified from the spectral fits (*n* = 9). **m**, f*b*_L_ was quantified from the absorbance of hemes *b*_L_ and *b*_H_ under each condition. All data are shown as the mean ± s.d. Due to one missing data point, a mixed-effects model with restricted maximum likelihood (REML) estimation was used, and fixed effects were analyzed using Dunnett’s multiple comparisons test to compare treatment to control. Source data

To determine f*b*_L_ from the reduction level of *b* hemes in the isolated mouse heart, transmural optical absorbance was measured by placing a white light-transmitting catheter inside the left ventricle (Fig. 3a). Transmitted light was collected using an integrating sphere to maximize signal by minimizing light loss due to scattering^36^ (Fig. 3b). Historically, the *b* hemes have been difficult to resolve and quantify in the isolated heart due to extensive myoglobin (Mb) absorbance over the same bandwidth (Fig. 1e)^32^. To overcome this limitation, we initially used myoglobin knockout (*Mb*^−/−^) mice, which are functionally similar to wild-type (WT) mice due to vascular compensatory mechanisms^37–40^. White light (Fig. 3c) was transmitted across the left ventricular wall, and the tissue absorbance was determined between 540 and 580 nm. Absorbance spectra (Fig. 3d) were fit with reference spectra representing reduced hemes *b*_H_ and *b*_L_ and cytochromes *c*_1_ and *c*, along with a linear component (Fig. 3e). The residuals, calculated as the difference between experimental spectra and best fits, were minimal, indicating a good fit (Fig. 3f**)**. To clearly visualize mitochondrial chromophores, the linear component was subtracted from the raw experimental spectrum and the best fit (Fig. 3g) after spectral fitting.

To determine whether the reduction levels of *b* hemes in the isolated heart respond as predicted to changes in ΔΨ_m_, and thereby whether this approach could be used to determine ΔΨ_m_, perfused hearts were challenged in series with interventions expected to alter ΔΨ_m_. Hearts were perfused normally for 10 min before the sequential addition of adenosine, KCl and carbonyl cyanide-*p*-trifluoromethoxyphenylhydrazone (FCCP). Adenosine is a vasodilator and mild negative chronotrope^41^ expected to suppress cardiac workload and improve oxygenation of the oxygen-limited heart, thereby increasing ΔΨ_m_ (ref. ^33^). Cardioplegia with KCl will eliminate contractile work and is thereby also predicted to elevate ΔΨ_m_, whereas uncoupling with FCCP will decrease ΔΨ_m_. Tissue absorbance was measured under each condition (Fig. 3h–j), and the absorbances of hemes *b*_H_ and *b*_L_ (Fig. 3k) and cytochrome *c* (Fig. 3l) were determined from the spectral fits. The parameter f*b*_L_ was then calculated (Fig. 3m). The changes in *b*_H_, *b*_L_ and f*b*_L_ in response to these cardioactive agents corresponded to the anticipated changes in ΔΨ_m_. The calibration curve generated in isolated mitochondria (Fig. 2n) was used to calculate ΔΨ_m_ from the f*b*_L_ values determined under each condition. In the isolated perfused *Mb*^−/−^ mouse heart, ΔΨ_m_ was 157 ± 14 mV (*n* = 9, mean ± s.d.) under control conditions. Adenosine decreased the heart rate from 297 ± 37 to 261 ± 46 beats per minute (bpm) and was associated with a slight increase in flow rate, whereas cardioplegia with KCl completely arrested the heart (0 ± 0 bpm; *n* = 9, mean ± s.d.). Adenosine increased f*b*_L_ to 0.59 ± 0.05 (*n* = 9, mean ± s.d.), and this was further increased following cardioplegia with KCl (0.62 ± 0.02; *n* = 9, mean ± s.d.). These values of f*b*_L_ exceeded the upper bound of the standard curve generated in isolated mitochondria, and thus, ΔΨ_m_ probably exceeded 208 mV under these conditions, the highest average value of ΔΨ_m_ we observed in isolated mitochondria. Uncoupling with FCCP decreased f*b*_L_ to 0.20 ± 0.04 (*n* = 8, mean ± s.d.), corresponding to a ΔΨ_m_ of ~101 mV.

### Cardiac ΔΨ_m_ during ischemia and reperfusion

A period of ischemia substantially alters mitochondrial function and is predicted to impact ΔΨ_m_. In particular, the precise kinetics and magnitude of ΔΨ_m_ repolarization following prolonged exposure to ischemia are unknown. Changes in ΔΨ_m_ upon reperfusion will affect the progression of tissue injury by influencing tissue energetics, ion distributions and mitochondrial ROS production. During ischemia, there is dramatic accumulation of the respiratory substrate succinate (Fig. 1b)^1,5,42^. Much of this accumulated succinate is oxidized within 1–2 min of reperfusion by the respiratory chain^42^, and this has been proposed to drive the return of ΔΨ_m_ to high levels shortly after reperfusion, potentially contributing to ischemia–reperfusion injury^1,5^. Our demonstration of real-time quantification of ΔΨ_m_ in the isolated perfused mouse heart allows us to address this key unresolved question in ischemia–reperfusion injury^1^.

**Fig. 4:**
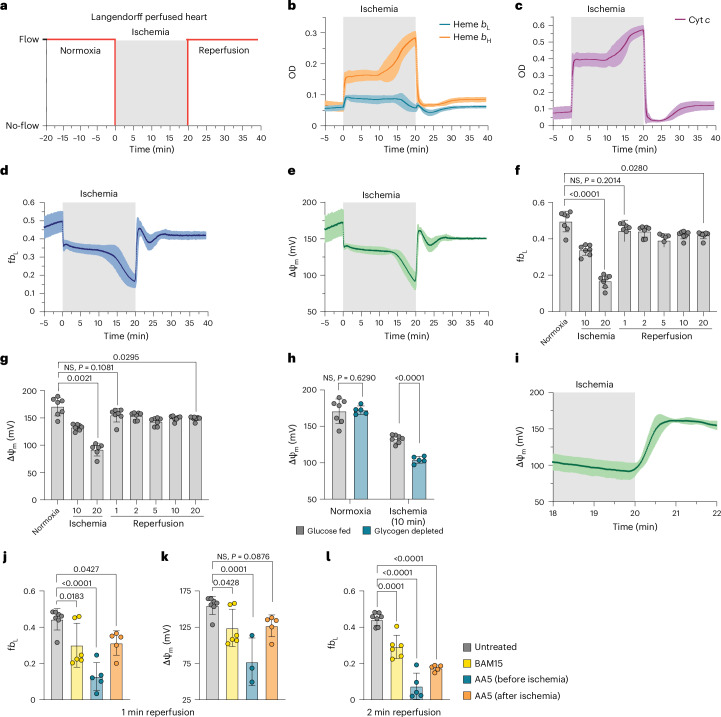
Changes in ΔΨ_m_ within the heart during ischemia and reperfusion. **a**, Schematic depicting the experimental protocol for global ischemia–reperfusion in the perfused heart. **b**,**c**, Hearts from *Mb*^−/−^ mice were subjected to global ischemia–reperfusion. Transmural absorbance was measured every second, and a rolling average of four consecutive spectra was taken before multi-wavelength nonlinear least squares regression analysis and quantification of hemes *b*_L_ and *b*_H_ (**b**) and cytochrome *c* (**c**) over time (*n* = 7). **d**, f*b*_L_ was quantified from the absorbances of hemes *b*_L_ and *b*_H_, and the average f*b*_L_ was plotted over time. **e**, ΔΨ_m_ was calculated from f*b*_L_ using the calibration curve generated in isolated mitochondria, and average ΔΨ_m_ was plotted over time. **f**,**g**, f*b*_L_ (**f**) and ΔΨ_m_ (**g**) were determined at control, 10 min or 20 min of ischemia and 1, 2, 5, 10 or 20 min of reperfusion in isolated hearts from *Mb*^−/−^ mice (*n* = 7). NS, not significant. **h**, Isolated perfused *Mb*^−/−^ hearts were perfused normally (*n* = 7) or with glucose-free KH buffer supplemented with sodium acetate and glucagon (*n* = 5). ΔΨ_m_ was determined just before ischemia (control) or after 10 min of ischemia. **i**, Panel **e** replotted to highlight ΔΨ_m_ over the first 2 min of reperfusion. **j**–**l**, Isolated hearts from *Mb*^−/−^ were subjected to global ischemia–reperfusion and treated with the uncoupler BAM15 (*n* = 6) at the onset of reperfusion or the SDH inhibitor AA5 before ischemia (*n* = 5) or after ischemia (*n* = 5). f*b*_L_ (**j**) and ΔΨ_m_ (**k**) were calculated after 1 min of reperfusion, and f*b*_L_ was again calculated at 2 min of reperfusion (**l**). All data are shown as the mean ± s.d. Statistical comparisons in **f**,**g**,**j**–**l** were made using one-way ANOVA with Dunnett’s multiple comparisons test. Multiple unpaired two-tailed *t* tests were performed with Holm-Šídák correction for multiple comparisons to compare substrate conditions in **h**. Source data

**Extended Data Fig. 1:**
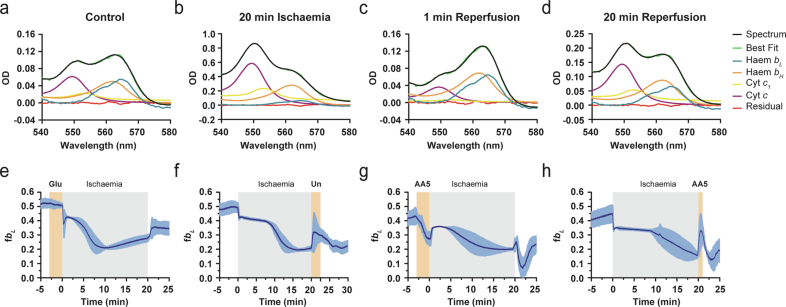
Measurement of ΔΨm in the Mb^−/−^ heart during IR injury. **a–d**, The isolated perfused Mb^−/−^ heart was subjected to global ischemia-reperfusion, and a multi-wavelength nonlinear least squares regression was performed on the experimental spectra collected under normoxic conditions (**a**), after 20 min of ischemia (**b**) and at 1 (**c**) and 20 (**d**) minutes of reperfusion using reference spectra for mitochondrial cytochromes and a line. After spectral fitting, the linear component was subtracted from all experimental spectra and best fits. **e**, Isolated perfused Mb^−/−^ hearts were perfused with glucose-free KH buffer supplemented with sodium acetate and administered glucagon (indicated by Glu) 3 min prior to ischemia (*n* = 5). Transmural absorbance was measured every second, and a rolling average of five consecutive spectra was taken prior to multi-wavelength nonlinear least squares regression analysis. Heme *b*_L_ and *b*_H_ absorbances were quantified, and f*b*_*L*_ was calculated over time. **f–h**, Isolated perfused Mb^−/−^ hearts received 2 µM BAM (*n* = 5) (indicated by Un) (**f**) at the onset of reperfusion or 20 nM AA5 (*n* = 5) (indicated by AA5) (**g**) 3 min prior to 20 min of global ischemia or 200 nM (*n* = 5) (indicated by AA5) (**h**) at the onset of reperfusion. f*b*_L_ was calculated over time as described above. All data are means ± s.d. Source data

To assess ΔΨ_m_ changes during ischemia–reperfusion injury, *Mb*^−/−^ mouse hearts were equilibrated under normoxic conditions and then subjected to 20 min of global ischemia, followed by 20 min of reperfusion (Fig. 4a). Tissue absorbance was recorded at 1 spectrum per second. Absorbance spectra between 540 and 580 nm were then fit with reference spectra of mitochondrial *b* hemes and cytochromes *c* and *c*_1_, which are shown for selected time points (Extended Data Fig. 1a–d). To make continuous measurements of tissue chromophores, we took a rolling average of four absorbance spectra and then performed spectral fitting on all spectra, which enabled us to quantify *b*_L_ and *b*_H_ absorbance continuously during our ischemia–reperfusion protocol (Fig. 4b). We also measured cytochrome *c* redox state over time as an indicator of mitochondrial oxygenation status, which showed rapid cytochrome *c* reduction at the onset of ischemia followed by reoxidation immediately upon reperfusion (Fig. 4c). From continuous measurement of *b*_L_ and *b*_H_ absorbance, we determined changes in f*b*_L_ and ΔΨ_m_ during ischemia–reperfusion (Fig. 4d–g).

At the onset of ischemia, ΔΨ_m_ rapidly declined to a new steady-state value of 133 ± 2 mV (mean ± s.d., *n* = 7), consistent with the lack of O_2_ preventing proton pumping by the respiratory chain (Fig. 4e–g). After 13 ± 2 min (mean ± s.d., *n* = 7) of ischemia, there was a further rapid decline in ΔΨ_m_, which reached a minimum of 92 ± 5 mV (mean ± s.d., *n* = 7) that coincided with cardiac hypercontracture (Fig. 4e–g)^36,43^. Hypercontracture is due to the sudden sarcomere shortening that occurs when the ATP/ADP ratio falls such that dissociation of the actomyosin complex is no longer possible, causing filament contraction^43^. Upon hypercontracture after about 10 min of ischemia, we observed an apparent increase in cytochrome *c* reduction (Fig. 4c). As previously described^36^, this change is consistent with an increase in the optical path length as the tissue contracts, rather than further reduction of the respiratory chain during ischemia. However, because both *b* hemes are impacted equally by changes in path length, f*b*_L_ is a normalized parameter and is unaffected by tissue geometry. We hypothesized that the presence of a relatively high ΔΨ_m_ during early ischemia could be explained in part by the glycolytic supply of ATP sustaining ΔΨ_m_ by reversal of the F_o_F_1_-ATP synthase^44–46^. Thus, inadequate production of ATP by ischemic glycolysis may explain the simultaneous occurrence of hypercontracture and the decrease in ΔΨ_m_ (ref. ^47^). A definitive test of this hypothesis was not possible in this system due to the poor delivery of oligomycin to the mitochondria, which would have enabled inhibition of reversal of the F_o_F_1_-ATP synthase. Even so, depleting hearts of glycogen before the onset of ischemia led to a more rapid onset of the decline in ΔΨ_m_, consistent with glycolytic ATP production sustaining ΔΨ_m_ early in ischemia (Fig. 4h and Extended Data Fig. 1e).

Upon reperfusion, cytochrome *c* was rapidly oxidized (Fig. 4c), consistent with the re-introduction of O_2_ driving respiratory chain activity and leading to the reestablishment of ΔΨ_m_ (Fig. 4e–g). The values of ΔΨ_m_ subsequently fluctuated before stabilizing after about 10 min of reperfusion. At 20 min of reperfusion, ΔΨ_m_ was significantly lower than before ischemia (Fig. 4g). These alterations in ΔΨ_m_ are likely to be in response to several factors that are changing during this time, including perfusate flow, calcium redistribution, oxidative damage, initiation of cell death pathways and induction of the mitochondrial permeability transition pore (MPTP), which will be explored in the future. Focusing on reperfusion, we observed rapid repolarization over the first 60 s of reperfusion (Fig. 4i), with ΔΨ_m_ reaching the same value as that at normoxia, before ischemia (Fig. 4g). This initial repolarization was blocked by the uncoupler BAM15 (Fig. 4j–l and Extended Data Fig. 1f), consistent with proton pumping by the respiratory chain driving repolarization. To further interrogate the contribution of succinate-driven respiratory chain proton pumping to the reestablishment of ΔΨ_m_, we pretreated the hearts with the irreversible SDH inhibitor atpenin A5 (AA5), which prevented ΔΨ_m_ hyperpolarization (Fig. 4j–l and Extended Data Fig. 1g). The addition of AA5 upon reperfusion also dampened repolarization, although this effect was smaller (Fig. 4j–l and Extended Data Fig. 1h). As SDH is a component of the citric acid cycle (TCA), the effect of AA5 may not be limited to succinate oxidation. Despite the variability observed in ΔΨ_m_ at 1 min of reperfusion following the addition of either BAM15 or AA5 after ischemia, we observed a progressive decline in ΔΨ_m_ between 1 min and 2 min of reperfusion (Fig. 4l) consistent with slow and variable uptake and subsequent target engagement of these hydrophobic compounds^48^ relative to the fast rates of repolarization (Fig. 4i).

**Fig. 5:**
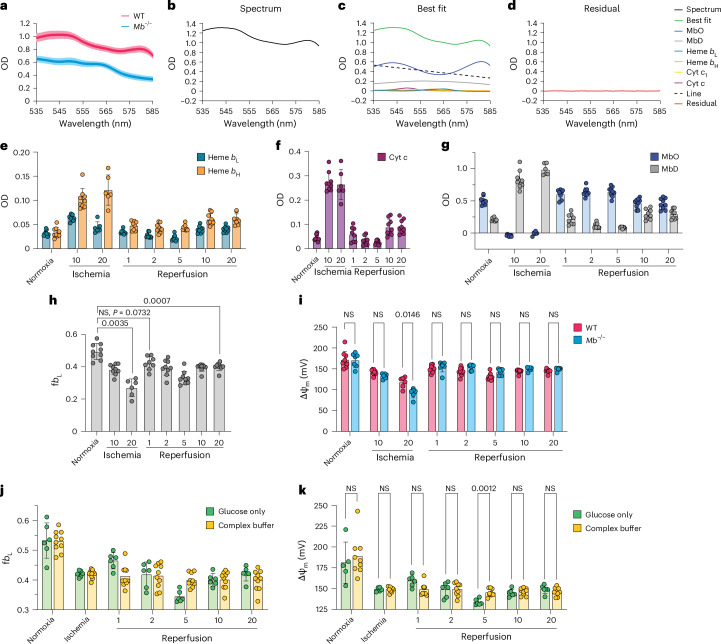
Changes in ΔΨ_m_ within the WT heart during ischemia and reperfusion. Isolated perfused C57BL/6N mouse hearts were subjected to global ischemia–reperfusion (*n* = 9). **a**, Average transmural absorbance of isolated perfused hearts from WT or *Mb*^−/−^ mice under control conditions (*n* = 3 each). **b**, Transmural absorbance was determined under normoxic conditions, and a multi-wavelength nonlinear least squares regression was performed using reference spectra for hemes *b*_L_ and *b*_H_ and cytochromes *c*_1_ and *c*, as well as MbO and MbD, and a line. **c**, The best fit and individual chromophore contributions. **d**, The residual of the best fit. **e**–**g**, The absorbances of hemes *b*_L_ and *b*_H_ (**e**), cytochrome *c* (**f**) and MbO and MbD (**g**) were quantified from the spectral fits at selected time points. Four data points from three biological replicates were excluded due to low spectral signal-to-noise ratio. **h**, f*b*_L_ was calculated as previously described. **i**, Comparison of ΔΨ_m_ at selected time points in isolated hearts from WT (*n* = 9) or *Mb*^−/−^ (*n* = 7) mice subjected to global ischemia–reperfusion. **j**,**k**, Isolated hearts from WT mice were perfused with KH buffer containing glucose only (*n* = 6) or glucose, lactate, pyruvate and butyrate (complex buffer) (*n* = 10) and subjected to global ischemia–reperfusion. Multi-wavelength nonlinear least squares regression was performed on the experimental spectra collected at selected time points, and f*b*_L_ (**j**) and ΔΨ_m_ (**k**) were calculated as previously described. Due to missing data points, a mixed-effects model with REML estimation was used in **h**, and fixed effects were analyzed using Dunnett’s multiple comparisons test to compare selected time points to control. Multiple unpaired two-tailed *t* tests with a Holm-Šídák correction were used to compare genotypes in **i** and substrate conditions in **k**. All data are shown as the mean ± s.d. Source data

**Extended Data Fig. 2:**
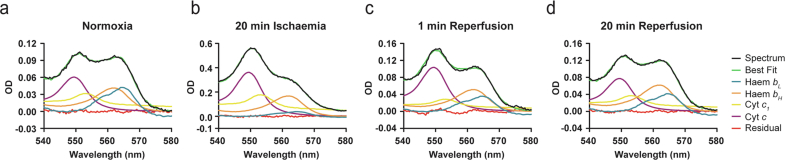
Multi-wavelength absorbance spectroscopy in the isolated Wt heart. **a–d**, Isolated perfused Wt mouse hearts (*n* = 9) were subjected to global ischemia-reperfusion, and a multi-wavelength nonlinear least squares regression was performed on experimental spectra collected under normoxic conditions (**a**), after 20 min of ischemia (**b**) and at 1 (**c**) and 20 (**d**) minutes of reperfusion. After spectral fitting, MbO, MbD and the linear component were subtracted from all experimental spectra and best fits. Source data

Having confirmed the feasibility of this approach in *Mb*^−/−^ mouse hearts, isolated hearts from WT mice were subjected to global ischemia–reperfusion. Hearts from WT mice had, as expected, greater absorbance than those from *Mb*^−/−^ mice due to Mb (Fig. 5a). Experimental spectra from WT hearts (Fig. 5b–d and Extended Data Fig. 2a–d) were deconvolved using reference spectra for oxygenated (MbO) and deoxygenated (MbD) Mb in conjunction with the mitochondrial cytochrome reference spectra used previously for the *Mb*^−/−^ hearts. In the case of the WT mouse heart, absorbance of *b*_L_ and *b*_H_ and cytochrome *c*, as well as MbO and MbD, was quantified from the spectral fits (Fig. 5e–g), and f*b*_L_ was determined at selected time points (Fig. 5h), rather than continuously due to the low signal-to-noise ratio observed in the WT heart. f*b*_L_ followed a similar profile during ischemia and reperfusion as in hearts from knockout mice (Fig. 5h), and indeed, ΔΨ_m_ values for WT and *Mb*^−/−^ hearts were the same under these conditions (Fig. 5i).

The above data were collected in WT hearts perfused with Krebs–Henseleit (KH) buffer containing glucose only. To evaluate the influence of substrate availability on the trends observed, we repeated these experiments in hearts perfused with glucose, lactate, pyruvate and butyrate and obtained similar results (Fig. 5j,k). Thus, we were able to quantify and characterize ΔΨ_m_ during ischemia and reperfusion in the isolated perfused heart. Together, these data show that maximal ΔΨ_m_ is rapidly reestablished upon reperfusion due to the restoration of respiration from carbon substrates in the presence of O_2_.

### Metabolic changes during ischemia and reperfusion

**Fig. 6:**
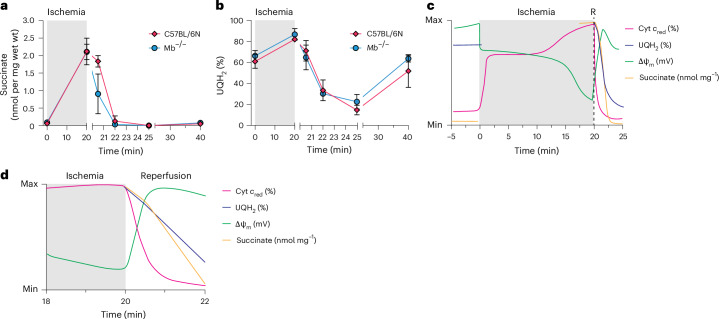
Metabolic changes during ischemia and reperfusion. Isolated perfused C57BL/6N or *Mb*^−/−^ mouse hearts (*n* = 6) were subjected to global ischemia–reperfusion. Whole hearts were rapidly frozen at control, after 20 min of ischemia or the indicated times after reperfusion. **a**, Succinate was extracted from frozen tissue samples and measured by LC–MS/MS against a [^13^C_4_]succinate internal standard. **b**, CoQH_2_ and CoQ were extracted from frozen tissue, and the percent reduction was determined by LC–MS/MS by determining the proportion of CoQH_2_ relative to total CoQ and CoQH_2_. **c**, Succinate and CoQH_2_ data from **a** and **b** were fit to interpolated curves and are plotted over time along with cytochrome *c* absorbance (Fig. 4c) and ΔΨ_m_ (Fig. 4i). **d**, Curves in **c** replotted to highlight reperfusion. The *y* axes of **c** and **d** represent the range of values observed in the heart in this study. Data presented in **a** and **b** are the mean ± s.d. Source data

We next investigated whether the energetic and metabolic status of the mitochondria during ischemia–reperfusion is consistent with oxidation of the succinate accumulated during ischemia by SDH upon reperfusion. The rapid hyperpolarization of ΔΨ_m_ upon reperfusion (Fig. 4i) and its prevention by the SDH inhibitor AA5 (Fig. 4k) are consistent with rapid oxidation of the accumulated succinate. However, it is also important to consider mitochondrial redox status, oxygen tension and respiratory activity. Therefore, we further interrogated how these other factors changed upon reperfusion of the ischemic heart. We found that cytochrome *c* was oxidized within 60 s of reperfusion (Figs. 4c and 5f). In the WT heart, Mb was reoxygenated within 60 s of reperfusion (Fig. 5g). Together, these findings are consistent with the rapid reoxygenation of the heart tissue and reestablishment of respiration, enabling proton pumping and the regeneration of ΔΨ_m_. These data also confirm that mitochondrial oxygen tension is sufficient for both respiration and the production of ROS early after reperfusion. Assessing the levels of succinate in the isolated perfused *Mb*^−/−^ and WT hearts during ischemia–reperfusion showed the expected accumulation of succinate during ischemia^5,49^, followed by its rapid return to pre-ischemic levels within 2 min of reperfusion (Fig. 6a). The rapid removal of succinate observed is consistent with its oxidation by SDH upon reperfusion, as well as some release from cardiomyocytes via the monocarboxylate transporter 1 (refs. ^50–52^). We assessed the redox state of the CoQ pool by measuring its percentage reduction by liquid chromatography followed by tandem mass spectrometry (LC–MS/MS)^53^, which showed that the CoQ pool became highly reduced during ischemia and was then oxidized back to normoxic levels within 1 min of reperfusion (Fig. 6b). Over the next 4 min, the CoQ pool became more oxidized than the control before becoming gradually more reduced over the subsequent 15 min (Fig. 6b). This observed change in redox poise of the CoQ pool upon reperfusion is consistent with its oxidation by complex III to facilitate proton pumping at complexes III and IV to support the reestablishment of ΔΨ_m_. Additionally, some CoQH_2_ may be oxidized by acting as a chain-breaking antioxidant to limit lipid peroxidation within the mitochondrial inner membrane^54^.

Combining these measures of mitochondrial function showed that they correlated over the crucial first minutes of reperfusion (Fig. 6c,d). Several important points emerged from these temporal comparisons. Respiration, and thus proton pumping by the respiratory chain, switched on within seconds as O_2_ was reintroduced into the ischemic heart, as indicated by the changes to cytochrome *c* and Mb absorbance. This activation of respiration correlated with the rapid increase in ΔΨ_m_ and with depletion of the accumulated succinate. The succinate oxidation was able to sustain a partially reduced CoQ pool over the first minutes of reperfusion. These findings confirm that ΔΨ_m_ repolarization occurs at the onset of reperfusion supported by succinate oxidation, consistent with a central contribution of a large ΔΨ_m_ to the initiation of ischemia–reperfusion injury.

## Discussion

The maintenance of ΔΨ_m_ is critical to mitochondrial function and cell integrity. Until now, it has not been possible to quantify ΔΨ_m_ in real time in the intact heart. Here, we exploited the biophysical properties of the *b* hemes of the cytochrome *bc*_1_ complex, which enabled us to relate the optical absorbance of hemes *b*_L_ and *b*_H_ to ΔΨ_m_. From the absorbance of *b* hemes, we generated a normalized parameter f*b*_L_ that was stable over a range of conditions and could be calibrated against ΔΨ_m_. Using this procedure, we were able to quantify ΔΨ_m_ in real time within the intact heart to investigate normal cardiac physiology and also to assess the impact of cardiac ischemia–reperfusion on ΔΨ_m_. This approach overcomes the many limitations associated with the use of exogenous probes, dyes and tracers because the *b* hemes are stably expressed and localized within the mitochondrial inner membrane. Additionally, electron exchange between the *b* hemes, and thus alterations in optical absorbance, occurs on the order of microseconds^55^, which enables close to real-time quantification of ΔΨ_m_. Many other in vitro or ex vivo models can be adapted to use this approach to assess how ΔΨ_m_ contributes to cardiac physiology and pathology.

Combining all measurements from WT and *Mb*^−/−^ hearts, ΔΨ_m_ was 166 ± 18 mV (*n* = 25, mean ± s.d.) in the nonworking, saline-perfused mouse heart under control conditions. How do these measures in the intact perfused heart relate to the mitochondrial Δ*p* in the heart in vivo? The low workload and somewhat restricted oxygen delivery of the saline-perfused mouse heart make it challenging to extrapolate these measurements to the in vivo working heart. Even so, after vasodilation with adenosine, ΔΨ_m_ surpassed ~200–210 mV, which may better reflect the in vivo condition where oxygen delivery is optimal, as evidenced by nearly complete Mb saturation^56,57^. Cardioplegia after vasodilation slightly enhanced f*b*_L_, suggesting that this condition approaches the maximal value of ΔΨ_m_ attainable. These maximum values of ΔΨ_m_ are higher than those usually reported for isolated mitochondria. This may be due in part to the fact that the retrogradely perfused heart is only doing rate work with no load, so the metabolic rate is more than tenfold lower than that with the maximum possible workload^58^. Thus, the unloaded heart that we interrogate here is much closer to state 4 than state 3. It is also important to note that preparations of isolated mitochondria are typically 0.8- to 1-µm spheres that arise from the shearing and resealing of the mitochondria present in tissues, which in the heart are primarily long, thin structures many micrometers in length^59^. It may be that the inner membranes of isolated mitochondria are thus more susceptible to proton leak than those of undisturbed mitochondria in intact tissue, possibly resulting in a more polarized maximum membrane potential in the perfused heart.

To determine Δ*p* (ΔΨ_m_ – 61.5 ΔpH), the magnitude of ΔpH (pH_cytosol_ – pH _matrix_) must be known; however, its value in the intact heart remains unclear. ΔpH amplitudes of –0.4 (ref. ^60^) and of –0.2 (ref. ^61^) have been reported in isolated mouse cardiomyocytes. These estimations suggest that ΔpH contributes 12–28 mV to the Δ*p*; however, further work is required to experimentally determine ΔpH values in the intact heart. Allowing for this gap in our knowledge, the in vitro ΔpH values give an estimate for Δ*p* of 178–194 mV in the isolated perfused heart. A similar extrapolation, with related caveats, suggests maximal values of Δ*p* of ~212–228 mV in the in vivo working heart.

The ability to measure ΔΨ_m_ in real time enabled us to assess how this parameter changes in a mouse heart model of global ischemia–reperfusion. Our analysis indicated that upon reperfusion of the ischemic heart, ΔΨ_m_ was reestablished to normoxic levels within 1 min, suggesting that the capacity to synthesize ATP by oxidative phosphorylation is rapidly restored. The reestablishment of ΔΨ_m_ correlated with the oxidation of the succinate accumulated during ischemia and maintenance of a partially reduced CoQ pool. These data are consistent with a model of ischemia–reperfusion injury in which mitochondrial superoxide production occurs at complex I by reverse electron transport (RET) at the onset of reperfusion^1,5^, initiating the oxidative damage that in turn leads to induction of the MPTP, cell death and the release of damage-associated molecular patterns (DAMPs) that activate the innate immune response. However, the timing and ordering of ROS production and induction of the MPTP are currently unresolved^14,15,17,50^. Future work will address this by assessment of ΔΨ_m_ over the several minutes of reperfusion along with parallel measurement of ROS production, mitochondrial Ca^2+^, pH and induction of the MPTP.

There are a number of considerations that potentially constrain the implications of this study that will be addressed in future work. Our approach assumes that the relationship between f*b*_L_ and ΔΨ_m_ is similar in vitro and in vivo and that the quantification of ΔΨ_m_ based on TPMP^+^ or TPP^+^ distribution is accurate. The optical approach using measurement of *b*_L_ and *b*_H_ provides a global measurement of the average ΔΨ_m_ in the mitochondria within the light paths interrogated, much like global ^31^P or ^13^C nuclear magnetic resonance studies. Therefore, this approach does not provide information about heterogeneous cellular responses to ischemia–reperfusion injury, which likely dictate individual cell fate; instead, these measurements reflect the average of all cardiomyocytes assessed. It should also be noted that the estimates of ΔΨ_m_ reported herein were obtained using a nonworking isolated heart model, which does not reflect the in vivo workload. Furthermore, accurate determination of the contribution of ΔpH to the value of Δ*p* is required. To do this will require the extension of real-time optical techniques to assess the pH of the cytosol and mitochondrial matrix under steady-state conditions and particularly during ischemia and reperfusion when the pH changes rapidly.

In summary, we have developed a method to quantify ΔΨ_m_ in real time within the intact, perfused mouse heart. We used this approach to determine the value of ΔΨ_m_ within the heart under normoxic conditions and then assessed the rate of reenergization of the mitochondria upon reperfusion, providing new insights into the contribution of the mitochondria to ischemia–reperfusion injury. This approach opens the way to assessing many other aspects of how the mitochondria contribute to cardiac pathophysiology.

## Methods

### Ethical approval

All animal protocols were approved by the Animal Care and Use Committee at the National Heart, Lung and Blood Institute, National Institutes of Health, Bethesda, USA, and were performed in accordance with the guidelines described in the Animal Welfare Act.

### Animal experiments

Male New Zealand white rabbits (Charles River) were singly housed with ad libitum access to food and water. Female and male mice were housed separately with ad libitum access to food and water. C57BL/6N mice (Taconic) were acclimatized for at least 7 days before experimentation.

### Mouse models

*Mb*^−/−^ mice were provided by C. Noguchi (National Institute of Diabetes and Digestive and Kidney Diseases)^40^.

### Isolation of mitochondria from rabbit heart

Male New Zealand white rabbits (6–12 months) received an intramuscular injection of ketamine (100 mg ml^−1^) and acepromazine (10 mg ml^−1^) followed by a mixture of medical air and O_2_ containing 3% isoflurane via inhalation. After confirming anesthesia by toe pinch, 1,500 units of USP-grade heparin was administered intravenously. Rabbits were killed by intravenous bolus of KCl (6 mEq), and the heart was rapidly excised and flushed with modified KH buffer containing (in mM) 137 NaCl, 10 HEPES, 5.4 KCl, 1.8 CaCl_2_, 0.5 MgCl_2_, 0.5 Na_2_HPO_4_, 10 glucose and 1 lactate (pH 7.4) and 1,000 units of USP-grade heparin. The mitochondrial isolation preparation procedure previously reported for dog and pig heart^58^ was modified as follows. The heart was dissected of atria, fat and connective tissue, and the remaining tissue was diluted to 20% (w/v) in cold isolation buffer containing (in mM) 10 HEPES, 5 K_2_HPO_4_, 1 EDTA, 1 EGTA and 280 sucrose (pH 7.1). The tissue was minced and diluted to 70 ml before mechanical homogenization (Virtis) on ice for 20 s at 40% power. Suspensions were digested with trypsin (0.5 mg per g tissue) for 15 min at 4 °C, and digestion was stopped with trypsin inhibitor (2.6 mg per g tissue) and 100 mg BSA. The suspension was centrifuged at 600*g* for 10 min at 4 °C, and the supernatant was collected. The pellet was resuspended with isolation buffer and homogenized using two passes of a 1-mm-clearance Dounce followed by five passes of a 0.2-mm-clearance Dounce. The homogenate was centrifuged at 600*g* for 10 min at 4 °C, and the supernatant was combined with the first isolate. The pellet was resuspended and pelleted at 600*g* twice more, collecting the supernatant each time. The pooled supernatant was centrifuged at 8,000*g* for 10 min at 4 °C, and the mitochondrial pellet was resuspended in isolation buffer and pelleted again at 8,000*g* for 10 min. The pellet was resuspended in Fiskum buffer containing (in mM) 125 KCl, 20 HEPES, 15 NaCl, 5 MgCl_2_, 5 K_2_HPO_4_, 1 EGTA and 1 EDTA, pH 7.1, and centrifuged once more at 8,000*g* for 10 min at 4 °C. This pellet was resuspended in Fiskum buffer and purified by Percoll gradient. The layer containing intact mitochondria was resuspended in Fiskum buffer and centrifuged at 13,000*g* for 10 min at 4 °C. The pellet was resuspended and centrifuged at 10,000*g* for 10 min at 4 °C. The final pellet was resuspended in 1 ml Fiskum buffer.

The fully reduced cytochrome *a* + *a*_3_ content was quantified assuming an extinction coefficient of 12 mM^−1 ^cm^−1^ for the reduced minus oxidized cytochrome *a* + *a*_3_ at 605 nm^62^. A 25-µl aliquot of mitochondria was added to 1 ml of 2% Triton X-100 buffer. An oxidized spectrograph was collected, and then 10 mM sodium dithionite was added followed by collection of a reduced spectrograph; the difference at 605 nm was determined, and the concentration of cytochrome oxidase was quantified. The average cytochrome *a* + *a*_3_ content in these preparations was 43.7 ± 1.5 nmol ml^−1^. Protein concentration was determined by Bradford assay (1856209, Thermo) using an albumin standard (23209, Thermo). The average protein concentration in these preparations was 23.9 ± 0.9 mg ml^−1^.

### Isolation of mitochondria from mouse heart

Mitochondria from eight C57BL/6N mouse hearts were isolated by mechanical homogenization and trypsin digestion and separated by differential centrifugation as previously described^63^ in isolation buffer containing (in mM) 225 mannitol, 75 sucrose, 5 MOPS, 0.5 EGTA and 2 taurine (pH 7.25). Cytochrome oxidase content was quantified as described above. Mitochondria (5–7 nmol cytochrome *a* + *a*_3_) were suspended in 5 ml Fiskum buffer containing (in mM) 125 KCl, 20 HEPES, 15 NaCl, 5 MgCl_2_, 5 K_2_HPO_4_, 1 EDTA, 0.6 EGTA and 0.25 CaCl_2_. Mitochondria were incubated sequentially with 5 mM GM, 0.5 mM ADP, 5 µM rotenone and 2 µM BAM15, and at least 50 samples were averaged for optical or TPMP^+^ uptake measurements.

### Mitochondrial absorption spectroscopy

A custom-made cylindrical glass chamber (15 mm in diameter × 50 mm in height) (Chemglass) was mounted to a scaffold attached to the lid of an integrating sphere (RTC-060-SF, LabSphere). A magnetic stirrer (SCS 1.11, Starna Spinette) was secured to the bottom of the scaffold, and the isolated mitochondrial suspensions were gently stirred. Light was supplied by a tungsten bromide light source and directed at the suspension at a 90° angle (LLC-10A, Lambda Scientific) via an optical fiber (FT1000UMT, Thor Labs). Transmitted light was focused to the bottom of the integrating sphere by three uniquely positioned baffles. Transmitted light was collected using an optical fiber (FT1000UMT, Thor Labs) connected to a cooled, rapid-scanning spectrometer (QEPro, Ocean Optics). The intensity of the light source without mitochondria present (*I*_0_) and the dark current of the spectrometer were collected at the start of each day. Transmitted light was recorded at 1,044 points between 348 and 745 nm at one sample per second using a custom LabVIEW-based program^32^.

### Isolation of cytochrome *bc*_1_ complex

Isolated rabbit heart mitochondria were diluted to 10 nmol cytochrome *a* + *a*_3_ per ml in isolation buffer containing 50 mM Tris and 1 mM MgSO_4_, adjusted to pH 8.45, at 4 °C. While mixing gently on ice, 10% (w/v) *n*-dodecyl β-D-maltoside (DDM) was added to 1% (w/v). The suspension was centrifuged at 40,000*g* for 40 min at 4 °C. The supernatant was transferred to a 2.5 × 20 cm glass column (Bio-Rad) containing DEAE Sepharose Fast Flow anion exchange resin (GE Healthcare) washed and equilibrated with isolation buffer C containing 0.02% (w/v) DDM. The column was eluted twice with ~4 ml isolation buffer C containing 0.02% (w/v) DDM before mitochondrial complexes were eluted by applying a linear NaCl gradient between 0 and 400 mM. The reddish-brown fractions eluted between 290 mM and 400 mM NaCl containing cytochrome *bc*_1_ were concentrated using an Amicon Ultra-15 centrifugal filter (Millipore) with a 10-kDa membrane cutoff and then diluted with 50% (v/v) glycerol and 0.1% lauryl maltoside.

### Collection of heme and cytochrome reference spectra

Reference spectra for hemes *b*_L_ and *b*_H_ and cytochromes *c*_1_ and *c* were collected in the integrating sphere. Purified cytochrome *c* (equine heart; C2506, Sigma-Aldrich) (1.5 nmol ml^−1^) was added to 5 ml Fiskum buffer, and the absolute absorbance spectrum of reduced cytochrome *c* was recorded after the addition of 5 mM sodium ascorbate.

To determine the absorbance spectrum of cytochrome *c*_1_, isolated complex III (7.1 nmol cytochrome *b* ml^−^^1^) was added to 5 ml Fiskum buffer. After recording the fully oxidized spectrum, 10 mM sodium ascorbate was added, and the absolute reduced cytochrome *c*_1_ reference was estimated by subtracting 66% of the oxidized absorbance to remove the spectral contributions from oxidized *b*_H_ and *b*_L_. The same sample was then titrated with 50 μl sodium dithionite (10% (w/v) in dH_2_O) to selectively reduce heme *b*_H_, as confirmed by the absence of both the peak at 566 nm and a shoulder at 558 nm that correspond to the *b*_L_ heme^34,35^. The *b*_H_ absolute reduced reference was obtained by subtracting the absolute cytochrome *c*_1_ reduced reference. This reference was determined to be accurate based on the negligible cytochrome *c*_1_ contribution in the Soret spectral region at 410 nm.

To collect the reference spectrum for heme *b*_L_, isolated mitochondria (2 nmol ml^−^^1^cytochrome *a* + *a*_3_) were added to 5 ml Fiskum buffer and made anoxic by incubating with 20 mM GM and 5 mM ADP while N_2_ was blown over the suspension. FCCP (0.5 μM) was then added to the suspension. The heme *b*_L_ reference was determined by taking the difference in absorbance between anoxia and after FCCP. The reference for heme *b*_L_ represents the difference between the reduced and oxidized species but is predicted to closely represent the shape of the absolute reduced absorbance spectrum, considering that the absorbance of oxidized heme *b*_L_ is nearly constant over this spectral bandwidth.

### Collection of myoglobin reference spectra

One C57BL/6N mouse heart was flushed with KH buffer containing 1,000 units USP-grade heparin and transferred into 50 mM HEPES on ice once fat, atria and connective tissue were dissected away. The heart was diluted to 20% (w/v), minced with scissors and then centrifuged at 10,000*g* for 10 min at 4 °C. The supernatant containing Mb was transferred to a 2-ml Eppendorf tube on ice. The absorbance of the oxygenated sample was measured between 400 and 750 nm (UV-2700, Shimadzu) before treatment with 10 mM sodium dithionite (10% (w/v) in dH_2_O) to deoxygenate.

### Spectral analyses

Spectra collected using a custom LabView-based program were converted to.csv files containing all wavelengths, *I*_0_, dark current and all transmission spectra. Optical data were analyzed using custom R scripts. Absorbance was calculated as the log_10_-transformed ratio of the incident to transmitted light. Absorbance data collected from isolated mitochondria or *Mb*^−/−^ mice were fit between 540 and 580 nm with reference spectra for hemes *b*_L_ and *b*_H_ and cytochromes *c*_1_ and *c*, together with a line, using a nonlinear least squares regression fitting routine. The model is described by equation (1) where *a–f* represent unique fitting coefficients. The fitted model describes the experimental absorbance spectrum as a combination of linear contributions from different spectral components and a linear component with variable slope and intercept.

1 $$\begin{array}{l}{\rm{Absorbance}}=({\rm{heme}}{b}_{{\rm{H}}}\times a)+({\rm{heme}}{b}_{{\rm{L}}}\times b)+({{\rm{cytc}}}_{1}\times c)\\ \,\,\,+({\rm{cytc}}\times d)+({\rm{wavelength}}\times e+f)\end{array}$$

Nonlinear least squares regression was conducted using routines for nonlinear least squares optimization in the minpack.lm package (https://www.rdocumentation.org/packages/minpack.lm/versions/1.2-4). Least squares analysis of the *bc*_1_ complex was previously shown to be superior to absorbance peak analysis^64^, including in the perfused heart^32,65^. To calculate the absorbance of individual hemes and cytochromes, the fit coefficient generated by the nonlinear least squares regression was multiplied by the optical density of the original reference spectrum at every wavelength.

### Determination of ΔΨ_m_ in isolated mitochondria

A TPP^+^-selective electrode (World Precision Instruments) filled with 10 mM TPMP^+^ and a reference electrode (MI-402, Microelectrodes) filled with 3 M KCl were conditioned in 100 mM NaCl. The TPMP^+^-selective and reference electrodes were placed into the mitochondrial suspension, and electrode voltages were measured with a pH millivolt meter (PH-1, Microelectrodes). Isolated mitochondria were incubated with 5 µM rotenone or without substrate for 15 min to de-energize mitochondria. The electrode was then calibrated in the presence of mitochondria by making six TPMP^+^ additions up to 2.17 µM. Electrode voltage was recorded at one sample per second. The changes in external TPMP^+^ were assessed and used to calculate the amount of TPMP^+^ sequestered by mitochondria, assuming a starting concentration of 2.17 µM and bath volume of 5.5 ml. The free concentration of TPMP^+^ was calculated by dividing the amount of TPMP^+^ taken up by mitochondria by the amount of mitochondrial protein added and then multiplied by the binding correction of 0.17 mg protein µl^−1^(ref. ^8^). ΔΨ_m_ was calculated by the Nernst equation: 61.5 log_10_ [TPMP^+^]_in_/[TPMP^+^]_out_ (mV).

### Studies of mitochondria isolated from rabbit heart

Mitochondria (2 nmol ml^−1^cytochrome *a* + *a*_3_) were added to Fiskum buffer supplemented with 0.6 mM CaCl_2_. O_2_ was gently blown over the surface unless otherwise noted. Mitochondria were incubated with 5 µM rotenone or starved before TPMP^+^ calibration as described above. Mitochondria incubated with 5 mM succinate (pH 7.0) were titrated with malonate up to 30 mM (pH 7.0) or 2 mM ADP (pH 7.0). To study the effect of pH, 1 µM nigericin was added to mitochondria incubated with 5 µM rotenone before TPMP^+^ calibration, or after the addition of succinate, and then titrated with malonate as described above. Mitochondria incubated with GM (pH 7.0) were similarly incubated with ADP until all ADP was converted into ATP (annotated state IV*). In succinate-fed mitochondria incubated with ADP, 10 mM malonate was added after state IV* to inhibit respiration. In GM-fed mitochondria, 5 µM rotenone was added instead. Mitochondria were uncoupled with 2 µM BAM15. To study anoxia–reoxygenation, mitochondria were incubated with 10 mM GM for 5 min before O_2_ was removed by gently blowing N_2_ over the surface of the suspension. Mitochondria were incubated under anoxic conditions for 5 min before O_2_ was reintroduced followed by 5 µM rotenone and 2 µM BAM15.

### Langendorff perfusion

Male or female C57BL/6N or *Mb*^−/−^ mice between 9 and 16 weeks of age were anesthetized (50 mg sodium pentobarbital) and administered 100 units of USP-grade heparin by intraperitoneal injection. After determining the depth of anesthesia, the heart was excised and submerged in ice-cold KH buffer with (in mM) 120 NaCl, 25 NaHCO_3_, 4.7 KCl, 1.75 CaCl_2_, 1.2 MgSO_4_, 1.2 KH_2_PO_4_ and 10 d-glucose that had been filtered (0.22 μm) before use. After excision, the heart was cannulated and retrogradely perfused at constant pressure (90 mm Hg) with KH buffer maintained at 37 °C and bubbled with 95% O_2_/5% CO_2_ (pH 7.4). The coronary sinus was not cannulated. For optical studies, the left atrium was removed, and a light catheter (FIP200220240, Molex) was positioned inside the left ventricular cavity. The heart was then secured in a water-jacketed glass chamber maintained at 37 °C. For tissue collection, the heart was submerged in a glass reservoir filled with KH buffer maintained at 37 °C. In all studies, the effluent flow rate was measured continuously using a flow-through sensor (1PXN, Transonic) placed before the heart, coupled to a Tubing Flow Module (TS410, Transonic). Temperature was measured using a flexible temperature microprobe (IT-18, AD Instruments). Flow and temperature signals were digitized via a PowerLab interface (AD Instruments). Flow was recorded at 1,000 samples per second, and heart rate was calculated based on the sinusoidal flow rate with the peak of each wave taken to be one beat and the number of peaks calculated over time.

### Myocardial optical absorption spectroscopy and analysis

The water-jacketed glass chamber harboring the isolated heart was positioned inside an integrating sphere, using a 6-inch-diameter integrating sphere (RTC-060-SF, LabSphere). Light was supplied by a 560-nm broadband white light source (LCS 0560-68-22, Mightex) powered by a BioLED Light Source Control Module (BLS-Series, Mightex). Transmitted light was collected using a multimode optical fiber (FT1000UMT, Thor Labs) fitted to the bottom port of the LabSphere and measured by a cooled, rapid-scanning spectrometer (VIS-A-S-100, Wasatch Photonics). The light intensity without tissue and dark current of the spectrometer were collected at the start of each day. Light intensity was recorded at 1,024 points between 400 and 800 nm at one sample per second using a custom LabVIEW-based program^32^.

R scripts described above were used to analyze optical data collected from either *Mb*^−/−^ or C57BL/6N (WT) hearts. Transmural absorbance data from WT hearts were fit between 535 and 585 nm with reference spectra for hemes *b*_L_ and *b*_H_, cytochromes *c*_1_ and cytochrome *c* and MbO and MbD, together with a line, using a nonlinear least squares regression fitting routine.

The signal-to-noise ratio was calculated by taking the sum of the calculated absorbances of hemes *b*_L_ and *b*_H_ and cytochromes *c*_1_ and *c* and dividing this by the sum-squared residuals. If this parameter fell below 100, the data points were excluded from analyses. A total of four data points from three biological replicates were excluded from analyses (Fig. 5e–i).

For one of the nine hearts in Fig. 3k–m, the addition of FCCP was associated with a dramatic increase in absorbance by heme *b*_H_ and cytochrome *c*, consistent with hypoxia due to respiratory uncoupling increasing O_2_ consumption beyond the amount of O_2_ available^33^. This outlier was omitted for the FCCP condition in Fig. 3k–m. This did not affect the statistical significance of the findings.

### Langendorff perfused heart studies

To assess cardioactive agents, hearts were perfused for 10 min before sequential administration of 15 µM adenosine, 11.3 mM KCl (final concentration 16 mM) and 2 µM FCCP. Adenosine and KCl were added directly to the buffer, and FCCP was infused at 1% of the coronary flow rate using a syringe pump (Pump 11 Elite, Harvard Apparatus).

To achieve glycogen depletion in the heart, glucose was replaced with 5 mM sodium acetate. Three minutes before ischemia, glucagon (G2044, Sigma-Aldrich) was infused at 10% of the coronary flow rate (final concentration of 2 µg ml^−1^)^66^. To confirm glycogen depletion, the levels of lactate in the heart were measured by LC–MS/MS after 20 min of ischemia. Lactate levels decreased by 66% in hearts perfused with acetate and glucagon. To study mitochondrial inhibitors and uncouplers during ischemia–reperfusion, DMSO stocks of BAM15 or AA5 were diluted in KH buffer and infused at 1% of the coronary flow rate to achieve the desired concentrations. BAM15 (2 µM) was delivered over the first 2.5 min of reperfusion. AA5 (20 nM) was administered 5 min before ischemia, or 200 nm AA5 was administered over the first minute of reperfusion; the higher concentration was required upon reperfusion due to slow uptake.

To assess the impact of substrate availability, hearts were perfused with KH buffer comprising (in mM) 118.4 NaCl, 25 NaHCO_3_, 4.7 KCl, 1.75 CaCl_2_, 1.2 MgSO_4_, 1.2 KH_2_PO_4_, 5 glucose, 0.2 sodium pyruvate, 1.2 sodium lactate and 0.2 sodium butyrate.

### Metabolite analyses

For metabolite assays, hearts were rapidly freeze-clamped using Wollenberger tongs, transferred to precooled Eppendorf tubes and stored at –80 °C. To assess succinate, frozen tissue (10–12 mg) was weighed into a precooled 2-ml, 2.8-mm ceramic Precellys tube (19-628-3, Omni) stored on dry ice. MS extraction buffer (25 µl mg^−1^ tissue) containing 50% (v/v) methanol, 30% (v/v) acetonitrile and 20% (v/v) H_2_O and 1 nmol [^13^C_4_]succinate internal standard (Sigma-Aldrich) were added to each sample, and samples were rapidly homogenized twice at 6,500 rpm for 15 s using a Precellys 24 tissue homogenizer (Bertin Instruments). Samples were incubated at –20 °C for 1 h and then centrifuged at 17,000*g* for 10 min at 4 °C. The supernatant was centrifuged at 17,000*g* for 10 min at 4 °C and then transferred to a 1.2-ml MS vial (29659, Supelco) on ice and stored at –80 °C. LC–MS/MS was performed using an LCMS-8060 mass spectrometer (Shimadzu) in conjunction with a Nexera X2 UHPLC system (Shimadzu). Samples (5 µl) were injected via a 15-µl flow-through needle, separated at 30 °C by a SeQuant ZIC-HILIC column (3.5 μm, 100 Å, 150 mm × 2.1 mm; Merck Millipore) with a ZIC-HILIC guard column (200 Å, 1 mm × 5 mm) at 200 µl min^−1^. Buffer A contained 10 mM ammonium bicarbonate, and buffer B contained 100% acetonitrile. A gradient of 80% buffer B was applied between 0 min and 0.1 min, 80–20% buffer B was applied between 0.1 min and 4 min, 20% buffer B was applied between 4 min and 10 min, 20–80% buffer B was applied between 10 min and 11 min and 80% buffer B was applied between 11 min and 15 min to achieve separation. MS was performed in negative ion mode, and multiple-reaction monitoring was used for specific detection of succinate to fragment transitions. Spectra were acquired and analyzed using LabSolutions software (Shimadzu). Compound quantities were calculated against a known concentration of the [^13^C_4_]succinate internal standard.

To assess CoQ redox state^53^, frozen tissue (3.5–5 mg) was weighed into a precooled 2-ml Precellys tube containing 1.4-mm ceramic beads (19-627-3, Omni) on dry ice and homogenized in 250 µl methanol and 250 µl hexane at 6,500 rpm for 15 s using a Precellys 24 tissue homogenizer (Bertin Instruments), transferred to new tubes and centrifuged at 17,000*g* for 5 min at 4 °C. Then, 20 µl of the upper hexane layer was transferred to a 1.2-ml MS vial (29659, Supelco), and the hexane was evaporated under a stream of N_2_ at 37 °C. Dried extracts were resuspended with 2 mM ammonium formate in 1 ml methanol, overlaid with argon and then stored at –20 °C before analysis by LC–MS/MS on the same day. Xevo TQ-S (Waters) was used for LC–MS/MS analyses of CoQ_9_ and CoQ_9_H_2_. Samples were loaded into the autosampler held at 8 °C, and 4 µl of sample was injected via a 15-µl flow-through needle. CoQ_9_ and CoQ_9_H_2_ were separated at 45 °C using an I-Class ACQUITY UPLCr BEH C18 column (2.1 × 50 mm, 130 Å, 1.7 µm; Waters) with a UPLC filter (0.2 µm; Waters). Methanol containing 2 mM ammonium formate was used for the isocratic mobile phase set to a flow rate of 0.8 ml min^−1^. The mass spectrometer was operated in positive ion mode. MassLynx 4.1 software was used to quantify the peak areas of CoQ_9_ and CoQ_9_H_2_, and the ratio of the peak areas was taken.

### Statistics and reproducibility

Data are reported as mean ± s.d. unless otherwise stated. A sample size of at least four biological replicates was used for mitochondrial studies and at least five biological replicates were used for heart studies, consistent with prior reports. Sample sizes for optical studies were validated with a power test for ANOVA using preliminary data. A priori power calculations were performed using the pwr package in R (version 4.4.0). Excluded data are described in the relevant Methods section. The experiments were not randomized, and investigators were not blinded during experiments or analyses. Statistical analyses were performed in GraphPad Prism 9, and statistical tests used are indicated throughout the text and in figure legends. *P* values < 0.05 were considered statistically significant.

### Reporting summary

Further information on research design is available in the Nature Portfolio Reporting Summary linked to this article.

## Supplementary information

Reporting Summary

## Source data

Source Data Fig. 1 Statistical source data.

Source Data Fig. 2 Statistical source data.

Source Data Fig. 3 Statistical source data.

Source Data Fig. 4 Statistical source data.

Source Data Fig. 5 Statistical source data.

Source Data Fig. 6 Statistical source data.

Source Data Extended Data Fig. 1 Statistical source data.

Source Data Extended Data Fig. 2 Statistical source data.

## Data Availability

Source data are provided with this paper.

## Code availability

The underlying code for this study is available from Zenodo at 10.5281/zenodo.17465842 (ref. ^67^).
